# Efficacy of a Multimodal Cognitive Rehabilitation Including Psychomotor and Endurance Training in Parkinson's Disease

**DOI:** 10.1155/2012/235765

**Published:** 2012-09-12

**Authors:** I. Reuter, S. Mehnert, G. Sammer, M. Oechsner, M. Engelhardt

**Affiliations:** ^1^Department of Neurology, Justus-Liebig University, Klinikstraße 33, 35392 Giessen, Germany; ^2^Department of Psychiatry, Cognitive Laboratory Justus-Liebig University, Am Steg 22, 35385 Giessen, Germany; ^3^Neurologisches Rehabilitationszentrum, HELIOS Klinik Zihlschlacht AG, Hauptstrße 2-4, 8588 Zihlschlacht, Switzerland; ^4^Department of Orthopedic Surgery, Klinikum Osnabrück, Am Finkenhügel 1, 49076 Osnabrück, Germany

## Abstract

Mild cognitive impairment, especially executive dysfunction might occur early in the course of Parkinson's disease. Cognitive training is thought to improve cognitive performance. However, transfer of improvements achieved in paper and pencil tests into daily life has been difficult. The aim of the current study was to investigate whether a multimodal cognitive rehabilitation programme including physical exercises might be more successful than cognitive training programmes without motor training. 240 PD-patients were included in the study and randomly allocated to three treatment arms, group A cognitive training, group B cognitive training and transfer training and group C cognitive training, transfer training and psychomotor and endurance training. The primary outcome measure was the ADAS-Cog. The secondary outcome measure was the SCOPA-Cog. Training was conducted for 4 weeks on a rehabilitation unit, followed by 6 months training at home. Caregivers received an education programme. The combination of cognitive training using paper and pencil and the computer, transfer training and physical training seems to have the greatest effect on cognitive function. Thus, patients of group C showed the greatest improvement on the ADAS-Cog and SCOPA-COG and were more likely to continue with the training programme after the study.

## 1. Introduction

Idiopathic Parkinson's disease (PD) is a neurodegenerative disorder characterized by loss of dopaminergic neurons and basal ganglia dysfunction. The prevalence of PD increases with age and is estimated in 100–200/100000 people [1, 2] worldwide. The clinical hallmarks of PD are akinesia, rigidity, and tremor [3, 4]. However, a spectrum of nonmotor symptoms occurs in Parkinson's disease. One of the most disabling symptoms is dementia, which is common among patients with PD with an average prevalence of 40% in cross-sectional studies and a cumulative prevalence approaching 80% [5, 6]. PD dementia is the third most common reason for dementia and is associated with rapid functional and motor decline, shortened survival [7], greater sensitivity to medication, higher risk of developing psychosis, reduced quality of life for both patients [8] and caregivers [9], increased caregivers' stress, and frequent transfer to nursing homes [10]. Clinical characteristics of PD dementia are cognitive slowing, executive deficits, visuospatial deficits, and memory impairments [11]. Pathological findings include Lewy bodies outside the substantia nigra, neurofibrillary tangles, and amyloid plaques [12]. Neurochemically, cholinergic deficits are found to be the most consistent associated with cognitive and neuropsychiatric symptoms [13].

In contrast to dementia, mild cognitive impairment (MCI) might occur early in the course of PD. Approximately, a quarter of PD-patients without dementia have mild cognitive impairment (PD-MCI). The Movement Disorder Society commissioned task force reported that MCI in PD is associated with increasing age, increasing disease duration, and disease severity [14]. However, 20% of PD-patients might have MCI at the time of diagnosis [15]. The clinical profile of PD-MCI is heterogeneous with a range of cognitive domains affected. Nonamnestic, single-domain impairment is the most common subtype of PD-MCI [14]. Criteria for the diagnosis of PD-MCI have been published recently [14]. Neuropsychological testing should include two tests within each of the five cognitive domains (attention, working memory, executive language, memory, and visuospatial). A diagnosis of PD-MCI impairment has to be found either in two neuropsychological tests in one domain or one impaired test in two different domains. Components of the executive systems are attention (focusing on relevant information), selective visual attention, inhibition (inhibition of irrelevant information) [15], overcoming of strong habitual responses or resisting temptation [16], task and time management, monitoring and coding of information for processing in the working memory, flexibility, set maintenance, and set shifting. The executive system can be viewed as a manager enabling the adaptation of the perceptive, cognitive, and motor system to new tasks [17]. Thus, patients with impaired executive functions face many difficulties in everyday life. They have a low attention span, difficulties in problem solving and decision making, in dual tasking, in set shifting, in visuospatial tasks, in adaptation to new tasks, and even in verbal learning and delayed recall. For example, PD-patients with impairment of executive functions may have difficulties in simultaneously driving a car and searching for a street or in keeping appointments. Executive dysfunctions also affect social components and the interaction with other people [15]. Patients are reported of being more irritable and having difficulties in suppressing inappropriate behaviour. PD-patients with MCI might have a higher risk to develop PD dementia. There is some evidence from previous studies that the presence of a nonamnestic single-domain MCI- subtype, executive deficits, impaired verbal fluency, visuospatial deficits, memory and language dysfunction predict PD-dementia, since patients with mild cognitive impairment have a higher risk of developing dementia. Intervention at an early stage of cognitive decline and prevention of the progress from MCI to dementia would be desirable. While treatment of motor symptoms has largely improved, treatment of cognitive dysfunction is still limited. Acetylcholine esterase inhibitors improve cognitive functioning only in some patients. Furthermore, transfer of improvements in cognitive training into activities of daily living has been extremely difficult. There is a large body of studies on animals and humans in the literature showing positive effects of exercise and sports on cognition [18–23]. Several studies suggest an enhancement of cortical plasticity by exercise [24, 25]. Executive functioning [19, 20, 26] as well as quality of daily living [27, 28] was found to be improved by aerobic endurance exercise. Patients, who complain of cognitive problems, suffer more often from cognitive deficits than patients without complaints [29]. Therefore, these patients should be offered neuropsychological testing. We have chosen a comprehensive training approach and designed a study using a multimodal cognitive training to improve cognitive functions. 

The aim of the present study was to compare the effect of a multimodal cognitive training regime including paper and pencil tasks combined with transfer tasks and a psychomotor training with a cognitive training based on paper and pencil tasks only and a cognitive training consisting of various tasks requiring executive functions combined with transfer tasks.

## 2. Methods

### 2.1. Subjects

240 patients, men and women who were between 50 and 80 years old and who had received a diagnosis of Parkinson's disease according to the UK brain bank criteria [4], were recruited for the study at the Hospital for Parkinson's Disease Bad Nauheim. Patients had been admitted at the hospital for rehabilitation. The presence of MCI was required for inclusion into the study. MCI was diagnosed when (a) patients complained of cognitive decline, preferably corroborated by a reliable source, (b) minimal effect on day-to-day functioning and the absence of dementia, and (c) presence of cognitive abnormalities which cannot be simply attributed to age. Exclusion criteria were severe concomitant diseases, which limit physical performances, a second neurodegenerative disease, and lack of motivation to improve cognitive deficits and the presence of dementia. De novo patients and those who had undergone surgery for DBS were not included into the study. All patients were assessed by a movement disorder specialist. Medical treatment was optimised prior to the study. It was aimed at keeping medication stable during the study. 

### 2.2. Design

The study was divided into two periods. Patients were enrolled into the study during an in-patient stay on a rehabilitation ward. The first part of the study (4 weeks) took place on the rehabilitation unit with a supervised cognitive training conducted by physiotherapists, occupational therapists, and two neuropsychologists. 

Patients were randomly allocated to one of the three training groups. Randomisation was conducted by using a computer-generated sequence. All groups received a cognitive training regime using paper and pencil material and a multimedial PC training. Group A received cognitive training only, while group B took part in a transfer training and a cognitive training. Group C conducted a cognitive training, and transfer and psychomotor trainings. Patients of group A and B had additional relaxation training and occupational training without transfer training to compensate for the additional training time of group C.

After randomisation medical history was taken, patients underwent medical and neurological assessments and were asked about their cognitive difficulties. They identified cognitive deficits that they want to improve. Severity of Parkinson's disease was assessed by using the Unified Parkinson's Disease Rating Scale (UPDRS) [30].

**Figure 1:**
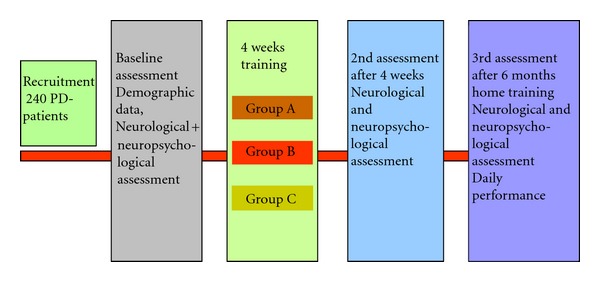
Study design group A: Cognitive training; group B: Cognitive + transfer training, group C: Cognitive, transfer + motor training.

Demographic data included information about age, body mass index (BMI), duration of disease, weekly sports activity, smoking habits, medication and concomitant diseases (hypertension, chronic obstructive pulmonary disease, thyroid disease, diabetes mellitus, hypercholesterinaemia, and osteoarthritis), education, profession, family, onset and severity of disease, history of psychosis, and impairments in daily living. Patients kept an activity log one week prior to the training programme and one week prior to the third assessment. Sports activities, time spent sitting, and doing light, moderate, and heavy work were recorded. The ethical committee of the Justus-Liebig University has approved the study and all patients have given written informed consent.  Figure 1 shows the study design.

### 2.3. Scales Used for Neurological and Neuropsychological Assessment of PD

For the assessment of the longitudinal course of the disease, the Unified Parkinson`s disease rating scale (UPDRS) was applied [30]. 

For the assessment of the goals of the cognitive training, the Goal Attainment Scale was chosen. The Goal Attainment Scaling (GAS) was used to define individual realistic and feasible goals according to patients' needs and expectations. The cognitive difficulties of the patients were assessed and the results of the baseline test were explained to the patients. In this study, GAS was measured using a 6-point Likert scale −3 represented function that is worse than at the start of treatment, −2 no change, −1 some improvement without meeting the expected goal, 0 represented goal achievement and +1 or +2 overachievement (exceeding the defined therapeutic goal) [31].

#### 2.3.1. Neuropsychological Tests

At the beginning of the study, all patients underwent two cognitive screening tests (PANDA and MMSE) [32, 33], followed by a detailed cognitive test battery including the ADAS-COG subscale and the SCOPA-COG as primary and secondary outcome measures, respectively. The neuropsychological assessment was repeated after the 4-week inpatient stay and after the 6-month training at home.

#### 2.3.2. ADAS-COG (Alzheimer Assessment Scale Cognition)

Although the ADAS-COG is the primary outcome measure in many clinical trials [34, 35], it is not a specific test for cognitive impairment in Parkinson's Disease. The scale was chosen as primary outcome measure in the current study, because it was the primary outcome measure in earlier trials assessing effects of medication on cognitive function in PD [6]. The study by Emre et al. [6] gave an estimate regarding the relevance of a 3-point improvement on the ADAS-COG in PD. Thus, it was possible to relate the results of the current study to previous results.

The conceptual framework underlying the ADAS-COG is to identify three reproducible factors: memory, language, and praxis [35]. The ADAS-COG score ranges in total from 0 to 70 points with higher scores indicating greater impairment. 

#### 2.3.3. SCOPA-COG (Scales for Outcome of Parkinson's Disease-Cognition)

The SCOPA-COG is an instrument which was designed to assess the specific cognitive deficits found in Parkinson's disease [36]. The scale consisting of 10 items covers the following domains: memory and recall (verbal recall, digit span backward, and indicate cubes), attention (counting backward, months backward), executive function (fist-edge-palm, semantic fluency, and dice), visual-spatial functions (assembly pattern), and memory (delayed recall). The score ranges from 0 to 43 points with higher scores reflecting better performance. 

#### 2.3.4. Additional Tests

Since PD-patients show pronounced deficits in executive functions additional tests for evaluation of executive functions were conducted at baseline, second and final assessment: Information processing speed (Paced auditory serial addition test (PASAT) [37]), Executive function (Behavioural assessment of the dysexecutive syndrome (BADS) [38]), Test for premorbid performance (Mehrfach-Wortschatz-Test (MWT-B) Multiple choice word test [39]), Assessment of mood and anxiety (Hospital anxiety and depression scale was applied [40]). 

#### 2.3.5. Health-Related Quality of Life (Parkinson's Disease Questionnaire 39 (PDQ-39))

For assessment of health-related quality of life, patients filled in the PDQ-39 [41]. It consists of 8 subscales. The sum score of raw data ranges from 0 to 156 points, with high scores indicating lower health-related quality of life. For better comparison of the results, raw data were transformed and expressed in percentages of maximal possible sum score.

The assessments were performed by psychologists and movement disorders specialists who were blinded to the treatment allocation of the patients. 

### 2.4. Training Programmes

Patients performed the training programmes in onstage and after optimisation of the medication. Dopaminergic deficits may affect the performance of patients in cognitive and physical exercises [42, 43].

#### 2.4.1. Cognitive Training

The cognitive training programme was individually tailored to patients' requirements based on the results of the baseline tests. Four individual (one-to-one) 60 min-lessons took place each week. All patients received at least 14 cognitive training sessions. 

The training included training of attention, concentration, biographical work, reasoning, memory, working memory, social rules, anticipation, cognitive information speed, prospective memory, cognitive estimation, problem solving, sequencing and planning, associations, and coping with disease.

For the training programme, a set of tasks requiring executive and memory functions was chosen from a variety of specific tests. Executive tasks of the BADS, which were not used for the baseline assessment, were included in the training. Simple patterns of the “Raven's Progressive Matrices” were used to establish problem solving strategies in the patients. Picture arrangement tasks, picture completion tasks, block design, and object assembly were adapted from the “Wechsler Intelligence test for children.” For improvement of verbal fluency, patients were encouraged to tell short stories or discuss short text passages. Photos were used for training of working memory. Tasks including visual search and rule finding were practised by using a PC-based programme. The training methods were designed to improve the various cognitive deficits, diagnosed at baseline, and focused on the executive functions. Task difficulty was adapted to the individual performance level of the patients.  Table 1 shows the content of the cognitive training and the percentage of time spent with different tasks.

#### 2.4.2. Transfer Training

The aim of the training was to support patients to manage their daily life better and to become more self-confident. Therefore, patients were asked to practise competence in tasks of daily routines. The transfer training programme was composed according to the baseline test results. Special preferences of the patients were considered. The transfer training included training of concentration, use of mnemonics, strategy (planning), navigational skills, impulse control, decision processes, listening training and memory, behaviour, calculating, handling of money, summarising of articles read or heard, and decision making. Typical tasks were to find the way to the supermarket or to prepare a meal, to go to the bank, pay a bill, and to use mnemonics. Patients had to look after a vegetable patch and some flowers to improve prospective memory. For better evaluation of the training, tasks were allocated to different categories: concentration, strategy, orientation, planning, and the use of mnemonic devices. The training took place 3 times a week, each lasted 90 min. Patients received at least 10 sessions of transfer training (Table 2).

#### 2.4.3. Motor Training

Group C performed a motor training resembling motor skill training or psychomotor training applied in children. The training included games and tasks designed to enhance inhibitory control, working memory, attention, visuospatial abilities, and planning and motor skills [44–47]. The training should also improve coordination, strength, speed, perception, and orientation. Patients should improve perception of their body. The therapeutic approach was based on individual capabilities and needs. Patients learned to perform motor sequences, dual tasking (walking and bouncing or throwing a ball), and spatial orientation tasks (finding items, remember hidden items). They walked through a parcours with obstacles following changing rules in order to improve anticipation and mental flexibility. For improvement of time estimation and movement performance mental imagery was used. Patients had to follow the guidance of the physiotherapist given by touch at different parts of the body. In addition, an aerobic training which should be of benefit for executive functions was performed. The training was conducted partly outdoors with inclusion of Nordic walking in summer. In winter patients walked on a treadmill. The training included at least 10, maximal 12 sessions each lasting 60 minutes. 

### 2.5. Education of Caregivers

A long lasting training effect depends on continuing training. Thus, cognitive training and exercises need to be adapted to the home environment. Accordingly, the caregivers most often the patients' family were included into the programme. The education for the caregivers consisted of 5 modules: information about Parkinson's disease, psychological aspects and the role of a caregiver, information about help aids, information on care management, assessment of individual problems, support in cognitive (all groups) and transfer training (groups A and B), NW and movement skill training. Course instructors were a specialist nurse, a physiotherapist and a psychologist.

**Phase II Continuation of Training at Home**

Corresponding to the allocation to the training groups patients got written instructions for the cognitive training, transfer training, and physical exercises at home. The instructions encompassed a collection of the tasks conducted during the stay at the hospital. Caregivers were advised how to organise the training but the hospital staff did not organise the training at home. All patients were asked to perform three 45 min cognitive training sessions per week using paper, pencil, and a computer programme. Patients of groups B and C were asked to continue with two transfer trainings per week and patients of group C got instructions to conduct movement skill training and aerobic training lessons twice a week. To compensate for the additional physical training of group C, patients of group A and B were provided with prescriptions for relaxation training.

### 2.6. Evaluation of the Training

All patients were tested using a neuropsychological test battery at three time points: prior to the training, and prior to discharge to assess the short-term effect, and 6 months after the training to assess the long-term effect. 

Caregivers were asked regarding their own well being and regarding the cognitive competence of the patients in activities of daily living. Patients and caregivers kept a diary to record training lesions. The diaries were collected and analysed at the 3rd assessment.

### 2.7. Statistical Analysis

Statistical analysis was conducted using IBM SPSS Statistics 18.0 (IBM, Somers, USA) statistical software. Formal power analysis was performed prior to the study. The power analysis was based on an improvement of the ADAS-COG by 3 points. The results indicated that a sample size of 60 subjects per group was sufficient. Since comprehensive training programmes including several assessments imply dropouts, a drop-out rate of 20% was taken into account. Demographic data on ordinal level were analysed by using a nonparametric test (Kruskall-Wallis). The Kruskall-Wallis test was also applied for the analysis of depression and the BADS subscales. Demographic continuous data were analysed by using One-way ANOVA. Linear model for repeated measures was used for analysis of training outcomes. The repeated measure analysis provides information about “between and within subjects” effects. Within subject effects give information about training effects over the assessment period. Linear trends showed if there was a systematic change of training effects over time. The interaction between groups and the linear trend of days (assessments) provided information about the difference in the rate of improvement between groups. The between subject factor compared the overall treatment effect between the groups. Post hoc analysis was done using Bonferroni tests. Parametric data were tested for normal distribution by using the Kolmogorov-Smirnov test. Significance level was set at 0.05.

## 3. Results

**General Results, Demographic Data and Accomplishment of the Training**

In total 223 patients (97.1%) completed the programme during the in-patient stay in the hospital: 71 (90%) patients in group A, 75 (93.8%) in group B, and 76 (95%) patients in group C. The patients were on average 64 ± 4 years old and that 8 years diagnosed with PD. The patients did not differ significantly in demographic data (Table 3). There was no difference in PD specific impairment and in the progress of PD between the groups.

Table 4 shows the percentage of time spent with different tasks. Patients of group A underwent on average 14.9 ± 0.7, patients of group B 14.7 ± 0.5, and patients of group C 14.8 ± 0.7 cognitive training sessions, respectively (*P* < 0.85). The time spent on different tasks was identical in all three groups.

Patients of group B conducted 11.2 ± 0.5 and patients of group C 11.4 ± 0.6 transfer training sessions (*P* < 0.9). 

Table 5 shows the percentage of time spent on different tasks of the transfer training, which was identical in both groups.

### 3.1. Motor Training

Patients had many difficulties to cope with the tasks. They struggled to find strategies to solve the tasks on their own, for example, they had difficulties to find the correct path through the parcours with obstacles and to follow the changing rules (set shifting). Patients tended to perseverate. Walking through a room with eyes closed only guided by different touches of the therapist challenged the patients as well since PD-patients have both deficits in proprioception and in perception of stimuli. The type of tasks and exercises were new to the majority of patients. PD-patients needed more time and repeated instructions to learn mental imagery. The lessons were conducted as individual lessons. It was not possible to conduct group lessons. About 40% of the training took place outdoors, 60% in the gym. 

### 3.2. Assessment of the Caregivers

The caregivers' knowledge about Parkinson's disease was assessed with a questionnaire at the end of the training programme. They were able to answer on average 27 ± 2.1 questions out of 30 compared to 20.2 ± 4.5 questions prior to the education programme. The carers' burden scale did not reveal any significant differences compared to baseline assessment. However, the questionnaire showed low burden in 60% of carers of group A, in 58% of group B, and in 62% of group C. Moderate burden was revealed in 30% of carers in group A, in 33% in group B, and 32% in group C. However, caregivers reported in a semi structured interview at the end of the education programme that they felt more confident. There was no difference between the caregivers of group A, B, and C. 

### 3.3. Training at Home

60% of patients of group A continued practising cognitive tasks 3 times a week for 45 min, while 40% conducted the training only once or twice per week. All patients of group B tried to continue the transfer tasks learnt during the rehabilitation, but further assessment showed that only 60% performed transfer tasks following a regular schedule. 75% of the patients of group B practised cognitive tasks 3 times a week. 90% of patients of group C pursued the training at home with the same quantity and intensity. They conducted a motor training three times a week, most often accompanied by their partners. The partners of patients of groups B and C managed to support their patients in practising transfer tasks. They asked them to prepare meals, to write the shopping list, or to go to the bank. 

### 3.4. Results of Neuropsychological Testing

The screening tests for cognitive functions did not reveal any differences between the groups and the results did exclude dementia. The neuropsychological baseline assessment did not reveal any differences between the groups either. Patients of all three groups had shown deficits mainly in tests addressing executive functions. Consecutively, the performance of the patients was worse on the subtests of the SCOPA-COG, semantic fluency, LURIA, dice and assembly pattern, zoo test of the BADS and PASAT. The memory tasks such as immediate and delayed recall were only mildly disturbed. 

The multiple-choice word test (MWT-B) was conducted as a measure for premorbid intelligence; the groups did not differ significantly either (*P* = 0.78). Thus, the randomisation process was successful.  Table 6 shows the neuropsychological test results over the course of the study.

### 3.5. Primary Outcome Measure

#### 3.5.1. ADAS-COG

All groups improved on the ADAS-COG significantly shown by a significant linear trend (*F*_*lin*⁡_[1, 220] = 150; *P* < 0.001). Group C improved most indicated by a significant interaction between groups and assessments (*F*_groups×assessments_[1,220] = 27.26; *P* < 0.001) and a significant group difference (*F*[2,220] = 7.7, *P* < 0.001). Further analysis showed that 78% of the patients showed some improvement at the second assessment, 51% of patients of group A, 85% of patients of group B, and 96% of patients of group C. 50% of the patients reached a reduction of the ADAS-COG score of 3 or more points, 18% of group A, 54% of group B, and 76% of group C. Six months after discharge of the rehabilitation unit 35% of patients (50% of patients of group A, 31% of patients of group B, and 28% of group C) showed a deterioration compared to the assessment at the end of the in-patient training programme. Further improvement was observed in 21% patients of group A, 37% patients of group B, and 50% patients of group C. 

#### 3.5.2. SCOPA-COG

**Figure 2:**
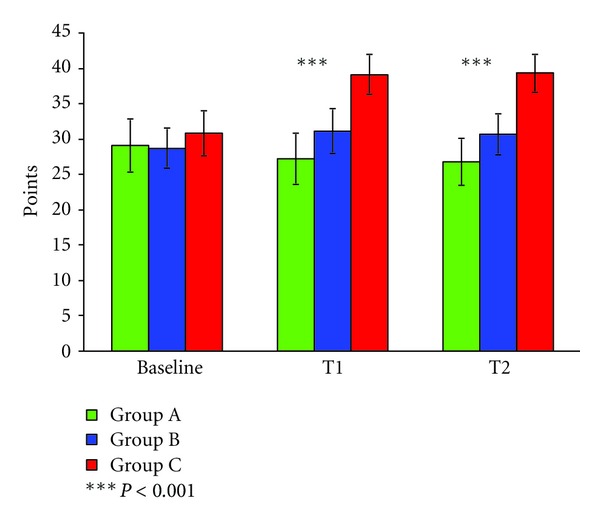
Group C improved significantly more on the SCOPA-COG test than group A and B.

In accordance the SCOPA-COG test showed a significant difference between the groups (Figure 2). All groups improved, indicated by the linear trend of days (*F*_*lin*⁡_[1, 220) = 46.09; *P* < 0.001). Group C improved most resulting in a significant difference between the groups (*F*[2, 220] = 31.4, df = 2; *P* < 0.001). Since the slopes of the improvements differed between the groups, a significant interaction between assessments and groups occurred (*F*_groups×assessments_[2, 220] = 65.63; *P* < 0.001). Post hoc tests revealed a significant difference between all groups (*P* < 0.001). Patients of group A reached 28.8 ± 3.7 points, group B 30.3 ± 2.7 points, and group C 37.6 ± 3.4 points. After completion of the in-patient training programme 31% of group A, 64% of group B, and 88% of group C had shown a significant improvement on the SCOPA-COG. Six months later at the final assessment 70% of patients of group A, 80% of patients of group B, and 94% of patients of group C had been able to keep their level of performance. Most improvement has been observed in the LURIA, dice, assembly pattern, and MOSAIC test of the SCOPA-COG. The pattern of improvement did not differ between the groups but the percentage of subjects showing an improvement and the speed of recovery.

#### 3.5.3. Executive Function

**BADS-Subscales**

The BADS-subscales especially the zoo map are a very demanding task requiring excellent planning skills. The subtests of the BADS (rule shift cards, zoo map, modified 6 elements test) showed the following results.

The baseline scores of the rule shift cards did not differ between the groups. There was a mild but significant difference between the groups at the second assessment (Chi-square = 7.1; *P* < 0.03) and final assessment (Chi-square = 9.1; *P* < 0.01).

At baseline assessment group C showed a tendency to perform better on the BADS zoo test. The mean profile scores of all groups were higher at the second assessment, but significant more patients of group C improved compared to groups A and B. There was a clear group difference at the second (Chi-square = 49.31; *P* < 0.03) and third assessment (Chi-square = 14.42; 0.001). At the final assessment six months after the discharge patients of groups A and B had lost most of the previously shown improvement. Only patient of group C managed to keep their level of performance. Thus, group C has been superior to groups B and A immediately after completing the training and at the second assessment six months later. 

There was no difference in the performance in the 6 elements test at baseline assessment. Group C showed a greater increase of the average profile scores leading to significant group differences at the second (Chi-square = 39.3; *P* < 0.001) and third assessments (Chi-square = 25.3; *P* < 0.001). 

#### 3.5.4. PASAT

The results of the PASAT test did not differ between the groups at baseline assessment, all groups produced on average 50% correct answers. Group A improved only marginally. Groups B and C benefitted from the training programme shown in a significant linear trend for assessments (*F*_*lin*⁡_[1, 154] = 63.71; *P* < 0.001). Since the improvement of the groups differed there was also a significant interaction between assessments and groups (*F*_groups×assessments_[2, 154] = 18.99; *P* < 0.001). Group C improved significantly more than groups B (*P* < 0.03) and A (*P* < 0.001) (*F*[2, 154] = 15.46; *P* < 0.001). 

Only 157 patients (group A: 50, group B: 53, and group C: 54) managed the PASAT test on the first assessment and were included in the statistical model. The other patients did not succeed in finding a strategy to cope with the task. On the second and third assessment 56 patients of group A, 64 patients of group B, and 71 patients of group C scored on the test. Groups B and C showed further improvement between the second and final assessment. 

#### 3.5.5. Goal Attainment Scale

The goals were identified at the baseline assessment. On the final assessment it was reviewed whether the goals were obtained. Patients of group C reached more often the main goal than the other groups (Chi-square: 57.1; *P* < 0.001). The detailed analysis of the results is shown in Tables 7 and 8.

The patients had selected the goals based on their self-evaluation of their cognitive impairment and on the advice given by the psychologist after the baseline testing. The main cognitive impairments reported by the patients could be attributed to the following domains: dual tasking, planning of complex and sequential tasks, decision making, rule recognition, rule shifting problems, delayed recall, and difficulties in finding misplaced items. 

Table 7 shows the goals patients had chosen and whether they were obtained at the final assessment. 

Planning of complex tasks, rule recognition, and shifting and dual tasking were identified as goals most often. While there was no significant difference between the goals chosen by the three groups there was a significant difference between the percentages of patients obtaining the goals between the groups. 71% of patient obtained the goal “decision making,” but only 40% of patients of group A.

Table 8 shows the improvement of the patients on the GAS scale with reference to their main goals.

More patients of group A compared to group B and C did not obtain the chosen goal or deteriorated compared to baseline, while 27.6% of patients of group C obtained the goal and 39.4% exceeded the expectations mildly and 7.6% substantially. The difference between the groups was significant (Chi-square = 48.23; *P* < 0.001).

### 3.6. Assessment of Mental State

15% of the patients in group A, 20% of group B, and 18% of patients of group C reported to suffer from depression and received medication. The results on the HADS depression scale indicated in 20% of patients of group A and group C, respectively, and in 25% of patients of Group B the presence of a mild to moderate depression. The anxiety level was assessed by using the Hamilton Anxiety Scale and did not differ between the groups. 

### 3.7. PD-Specific Impairment at the Final Assessment

**Figure 3:**
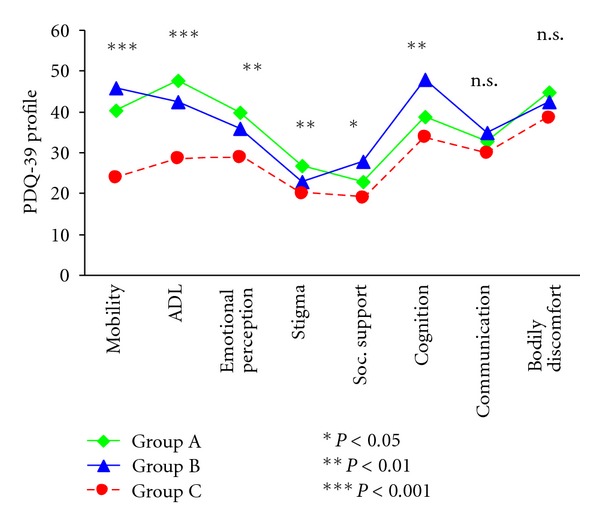
PD-patients of group C reported less PD-specific impairment at the final assessment. The *y*-axis shows the percentage of the maximal possible sum scores. The *x*-axis shows the 8 subscales of the PDQ-39. The lines represent the scores the groups have achieved in the 8 subscales.

The results of the PDQ-39 at the final assessment showed that patients of group C rated their health-related quality of life higher than the other groups. 13.8% of patients of group A, 38% of patients of group B, and 52% of patients of group C reported less impairment due to PD (Figure 3). 

### 3.8. PD-Specific Functioning

The UPDRS score showed a mild improvement in all groups at the final assessment but there were no significant differences between the groups indicating that the cognitive improvement could not be referred to a nonspecific effect resulting from general physical improvement (Table 9).

**Performance in Activities of Daily Living at the Final Assessment and Evaluation of the Training Programme by Patients and Caregivers**

The patients of group C reported that they had adapted a more active life style, felt more confident in activities of daily living, and had taken over some more chores. They perceived their partners and caregivers as being helpful. They enjoyed the participation of their partners in conjoint sports activities.

Patients of group B also regarded the training programme as helpful but reported of having still problems with activities of daily living. Patients of group A had more difficulties with transfer of skills into daily life and the carryover effect was smaller than in the other groups. 

65% of the caregivers of patients in group C, 54% of caregivers of group B, and 49% of caregivers of group A found that competence and cognition of their patients had improved in activities of daily living. A deterioration of the performance in daily living was reported in 11% of group C, 17% of group B, and 25% of group A. 

Caregivers felt more relaxed and competent to manage difficult situations, while patients accepted the guidance of their caregivers better than prior to the training. Caregivers felt confident to support the partners with the training at home. 

Patients of group A felt that the cognitive training was arduous at times. Some patients perceived the training as stressful. Patients of groups B and C were asked to compare the training programmes. Patients of group C preferred the motor training to transfer training and cognitive pencil and paper tasks. 80% of patients judged the training as strenuous and felt sometimes exhausted. 30% of patients reported of being frustrated at times but did not ask for help or further explanations. 

In summary patients who conducted a multimodal cognitive rehabilitation programme improved most on the ADAS-COG and SCOPA-COG, reported a better quality of life, were more active, and continued coping with daily tasks. Patients of group C were physically more active after the training programme and a higher percentage of patients of group C continued with the cognitive training. Physical improvement did not explain the difference in cognitive performance after the end of the study.

## 4. Discussion 

Dementia is a part of the Parkinson's disease spectrum. Currently, there is no treatment aimed at halting or reversing disease progression [6]. Most treatment approaches are based on substituting neurotransmitter deficits. However, the effect of the drug treatment is limited and inconsistent among PD-patients. Therefore, other treatment options are needed. The current study has shown that a multimodal cognitive training programme might improve cognitive performance in PD-patients with mild cognitive impairment. The effect of the multimodal cognitive training on cognitive functioning was comparable to the effect found by Emre et al. in the rivastigmine trial [6]. The superiority of group C with respect to the cognitive assessments suggests that a diversified and challenging training is more effective. The results support our hypothesis that a special motor training might positively affect cognitive functioning. In children movement skill training is used to improve working memory, inhibition of impulses, attention, and visuospatial capacity. The expression psychomotor training is often used in Germany for physical training that combines physical and cognitive tasks. Oswald et al. [46, 47] were the first who called the physical training conducted in the SIMA project psychomotor training. They found that a combined psychomotor and memory training led to an improvement of psychomotor performance and to an improvement of cognitive performance and competence. Neither the psychomotor training nor the memory training on its own resulted in such effects. Oswald et al. [46, 47] assumed that neurophysiological changes led to a provision of reserve capacity of CNS performance. Therefore, emphasis should be placed on the reduction of cognitive load in neuropsychological training programmes. Goebel et al. [48] postulated as well to reduce instructions and working memory load during the training and to use procedures which lead to a more automated, implicit strategy application which demand less executive control [49–51].

Oswald et al. [46, 47] also suggested that the memory training affects psychomotor performance confirming the “memory-movement-hypothesis.” An interaction between motor training and memory training might support remembering movement sequences. Our results confirm the hypothesis of Oswald et al. [46] that more diverse training programme enhances motivation and resulted in more regular training. Patients of group C exercised more physically and conducted more paper and pencil lesions at home than the other groups indicating that the attitude towards learning was positively influenced. In addition, the partners of patients in group C participated actively in the sport programme which helped patients to adhere to the training programme. As known from questionnaires we had sent to patients and their families inquiring about the training; social aspects are very important for PD-patients. Home-based multimodal cognitive training programme was sufficient to keep the performance of the second assessment in patients of group C. The psychomotor training combined with endurance training might have contributed to the superiority of group C. There is a huge body of literature suggesting a prevention of cognitive decline by life long exercise or even an improvement of cognitive deficits by physical activity. Executive functions may be selectively maintained or improved in people with better physical condition provided by physical training [52]. The importance of aerobic physical exercise on cognitive functions, especially on executive functions, has been shown [19–21, 26]. The studies have been mainly conducted in healthy elderly or patients with dementia but older people with PD can benefit their executive functions in the same way, as do their peers without PD. The results of some studies have shown that brain areas undergoing biological aging benefit most from endurance sports. Even structural changes have been observed [21]. Since group C conducted more training lessons at home compared to the other groups, one might argue that the superiority of group C was rather due to the quantity of the training than to the content. However, group C has already performed better at the second assessment and group A had shown poorer results than groups B and C. At this time all groups spent a similar amount of time with training and received similar attention by the therapists. Thus, the content of the training might be responsible for the different performance of the groups. The superiority of group B compared to group A suggests the efficacy of the transfer tasks. The psychomotor training helped group C to improve further, especially in the challenging executive tasks regarding rule cognition, set shifting, and decision making. The authors had also taken care during the study design that groups B and A received relaxation training and physiotherapy as compensation for the motor training of group C during the 6-month training period at home. These treatment offers were also accepted by the patients but especially patients of group A did not practise cognitive lessons at home as much as they were advised to do. Home-based cognitive training without transfer and physical training as performed by group A was less attractive for the patients. 

The groups managed the executive tests very differently. The BADS zoo map is a very challenging test as mentioned above and requires various training approaches to achieve an improvement. Only patients of group C obtained a permanent improvement on this test.

Thus, the different training schedules affect the training outcome also regarding the type of tasks, which were better performed. Patients of group C managed executive tasks better than group A and B, which is shown by the better performance on the BADS test battery and the better results on the SCOPA-COG. Exercise and endurance training is thought to improve executive functions most. Furthermore, brain areas which are more vulnerable for age-related volume lose benefit most from physical exercise. 

Patients with mild cognitive impairment benefit more from cognitive training than more advanced patients. Studies have shown that the effect of physical exercise on demented patients is limited. Patients have to be capable of understanding the training programme. Depression might also influence the performance in neuropsychological tests. Klepac et al. [53] have found that depression preceding PD motor signs might favour poorer cognitive abilities. However, there was no significant difference between the groups regarding the percentage of patients being depressed and the severity of depression. Thus, depression and anxiety were not confounding factors and not responsible for the different treatment outcomes of the three groups. Assessment bias in favour for one treatment can be excluded because the movement disorder specialists conducting the tests were blinded to the treatment arms. 

The findings of the study confirmed as well that PD-patients benefit from a specific cognitive training. Based on the results of a previous study [54], we had chosen an individual training approach. Therefore, patients did not undergo a standardised cognitive training programme but a programme tailored to their needs. Interestingly, the results of the baseline testing and the psychologist's training suggestions were very often concordant with the requests of the patients. Therefore, the goal attainment scale was very suitable to represent both the training suggestions and the patients' requests. The fact that more patients of group C attained the selected goals might also support the strength of a multimodal training. Resembling the results of the executive tests some goals seemed to be more difficult to attain (see Tables 7 and 8). Patients struggled more to attain goals such as rule generation or rule shifting while dual tasking and memory improvement were easier to accomplish. However, group C was also more successful in achieving an improvement in planning of complex tasks, rule generation, rule shifting, and decision finding than groups B and C. 

In contrast to a study by París et al. [55], the present study suggested a translation of improved cognitive performance into daily living. The patients' caregivers also reported an improved competence in real life. However, patients practised during the transfer training situations of everyday life. Hence, they did not face completely new situations in their daily life. París et al. [55] found an improvement in attention, information processing speed, memory, visuospatial and visuo-constructive abilities, semantic verbal fluency, and executive functions but no improvement of cognitive difficulties in daily living. 

Sinforiani et al. [56] observed a carryover effect after completion of a 6-week cognitive rehabilitation training including cognitive and physical training. The authors suggested that the combined cognitive rehabilitation training exerts its positive effects by reinforcing cognitive strategies with improvement of frontal lobe functions.

Quality of life is closely associated with nonmotor symptoms of PD, especially cognitive function [8]. Consequentially Group C scored much higher on the PDQ-39 than the other groups. Thus, health-related quality of life was improved markedly in these patients. In contrast to our results París et al. [55] did not find any benefits of the cognitive training in self-reported quality of life. Probably, the cognitive training has to lead to improvements in daily living in order to improve quality of life. 

Research over the last decade has shown that cognitive deficits affect motor performance. Patients with cognitive deficits had more difficulties in motor tests than patients without cognitive deficits. Hausdorff et al. [57] have found a close correlation between walking and executive functions. Yogev et al. [58] have shown that gait variability in dual tasking is closely associated with the performance in executive tasks. The difficulties that patients of group C experienced while solving the motor tasks have been in accordance with these results. Nevertheless, most of the patients managed to cope with the training programme, though some patients felt overstrained and quitted the programme. 

### 4.1. Limitations of the Study

One might criticise that we compared three different treatment arms and did not include a control group without cognitive training in this study. However, patients were enrolled into the study during their stay on a rehabilitation unit and complained of a deterioration of their cognitive performance. For this reason, it was not possible to withhold treatment. Further, we had shown the superiority of a cognitive training compared to standard treatment in a previous study [54].

Another limitation is that there are no evidence-based data for the transfer training. Further research is necessary to evaluate and validate which transfer exercises are useful tools. The psychomotor training or movement skill training has been used for many years in children and has been applied in patients with dementia [53, 54]. However, it has not been validated in PD-patients so far. The selection of tasks has been based on the experience of the therapists and medical staff and published data which were based on the work with children. 

One might also argue which improvement on the ADAS-COG or SCOPA-COG might be clinically relevant. However, the scales are validated and had been often applied in clinical trials. The clinical relevance of the improvements has also been shown by the observed translation into real life. Furthermore, due to the short follow-up period of 6 months we cannot report on long-term effects. However, studies assessing long-term results are very difficult to conduct since it is very difficult to keep the medication stable. 

### 4.2. Strengths of the Study

To our knowledge, the results of the current study show for the first time, that a multimodal cognitive training in patients with Parkinson's disease can lead to improvements of cognitive function and improves quality of life. In addition, there was some translation of the cognitive improvement into real life. We also want to emphasize that it was a blinded randomised study and the drop-out rate was low. 

## 5. Conclusion

In conclusion, we have shown that PD-patients with cognitive deficits benefit from a multimodal cognitive training. The multimodal training was superior to a paper- and pencil-based cognitive training. The combination of the cognitive training with a motor training seems to be most successful and the short-term effect of the training on cognitve functions was comparable to the effect found in the rivastigmine trial. However, we cannot predict the long-term effect of the cognitive multimodal training. The study has shown some translation of cognitive improvements into “real” life. Admittedly, the multimodal training of cognitive functions is time consuming, requires high motivation of the patients, and put demands on resources. Due to the quantity and quality of the trainings sessions it will also be costly. On the other hand dementia is a risk factor for falls, high morbidity and transfer to nursing homes, which increases the costs for the patients' care substantially and jeopardizes the patients' quality of life. 

## Figures and Tables

**Table 1:** Cognitive training.

Training section	Examples for tasks
Planning strategies	Shopping lists, key search of the BADS subscales, to get items out of a bottle, special wooden 3-dimensional jig saw
Mnemonics	Using memory hooks, writing of suitable mnemonics, and planning where to place them
Decision making	Computer tasks, painting
Set shifting	To solve various tasks according to changing rules, to calculate for 5 min, then to read for 10 min and to write for 5 min, or to switch tasks after every third answer, categorising items according to different rules
Calculation	Calculation tasks
Navigational skills	Finding the way through a maze on a screen, finding the way around obstacles on a paper or on a screen
Information speed processing	Connecting numbers or letters, connecting figures to an image, and finding the meaning of a fictive word
Summary	Extracting the relevant information from news or from a story or a short movie
Concentration	Finding similar or dissimilar items

**Table 2:** Transfer training.

Tasks	Examples
Planning and sequencing	Preparing of meals, shopping, construction of items, repairing of items, and art works
Reasoning	Finding the right solution, using deductive strategies, finding a way by incomplete instructions, to find out how things work, and use of unfamiliar tools
Concentration	Sorting and selection tasks, to fit small items, to build a model of a castle, knitting and weaving patterns, jig saw, to listen to a story and to press a button, when a previously identified word was read
Memory	Role play, music performance, singing, and learning poems
Working memory	Games, n-back tasks, dual tasks like driving on a driving simulator and listening to the news, calculating during walking, to follow complex instructions, and to extract the necessary information out of a long text
Anticipation	Walking on a crowded sidewalk, watching a movie and predict what people will do next, finish a picture story, and decipher the mood of people on pictures
Pospective memory	To look after a vegetable patch and flowers, to keep appointments, and to take over special tasks at the beginning of the week
Cognitive information speed	Follow verbal instructions given with increasing content of information and speed, card games, and complex reaction tasks
Social rules	Define the appropriate behaviour in different situations, role play,
Association	Solving tasks by association, finding common features, and categorisation
Cognitive estimation	Estimation of height, quantity of items shown, and weight

**Table 3:** Demographic data.

	Group A			Group B			Group C		
	*N* = 71			*N* = 75			*N* = 76		
Gender	F = 35		M = 36	F = 36		M = 39	F = 36		M = 40
Duration of PD (months) *∅* ± SD		98 ± 8			95 ± 9			100 ± 6	
Stage (Hoehn and Yahr)									
II		*N* = 7			*N* = 6			*N* = 10	
III		*N* = 55			*N* = 59			*N* = 58	
IV		*N* = 9			*N* = 10			*N* = 8	
Medication									
L-Dopa		Yes: *N* = 68			Yes: *N* = 64			Yes: *N* = 59	
Dopamine agonist		Yes: *N* = 53			Yes: *N* = 56			Yes: *N* = 59	
MA0 inhibitor		*N* = 43			*N* = 38			*N* = 43	
COMT inhibitor		*N* = 33			*N* = 31			*N* = 34	
Antidepressants		*N* = 7			*N* = 8			*N* = 8	
Neuroleptic drugs		*N* = 5			*N* = 8			*N* = 7	
Formal education (years) *∅* ± SD		10 ± 1.2			11 ± 0.6			11 ± 1.0	
Marital status m = married, s = single, and p = partner	m = 58	s = 9	p = 5	m = 61	s = 11	p = 3	m = 63	s = 9	p = 4
Home (own home, renting)	Own: *N* = 40		Renting: *N* = 32	Own: *N* = 43		Renting; *N* = 32	Own: *N* = 40		Renting: *N* = 36
BMI *∅* ± SD		27.5 ± 4			26.8 ± 7			27.2 ± 3	
Smoking	Yes: *N* = 7		No: *N* = 65	Yes: *N* = 10		No: *N* = 65	Yes: *N* = 9		No: *N* = 67
Sports activities (min)/week *∅* ± SD		155 ± 17			163 ± 25			147 ± 17	
Physical work h/week *∅* ± SD									
Very hard		8.5 ± 2.6			9.2 ± 2.8			9.8 ± 2.1	
Hard		15.5 ± 4.5			14.9 ± 5			15.1 ± 5.5	
Comorbidity									
Coronary heart disease		*N* = 7			*N* = 6			*N* = 8	
Hypertension		*N* = 32			*N* = 33			*N* = 36	
Diabetes mellitus		*N* = 7			*N* = 10			*N* = 8	
COPD		*N* = 5			*N* = 6			*N* = 9	
Thyroid disease		*N* = 12			*N* = 10			*N* = 11	
Hypercholesterinaemia		*N* = 36			*N* = 32			*N* = 27	
Osteoarthritis		*N* = 27			*N* = 31			*N* = 34	

Number of patients is shown as total number, mean values are shown *∅* ± SD.

**Table 4:** Distribution of cognitive training.

Training section	Percentage of time spent (%)
Planning strategies	17
Mnemonics	15
Decision making	15
Set shifting	15
Calculation	10
Navigational skills	10
Information speed processing	8
Summary	5
Concentration	5

**Table 5:** Transfer training: time spent on different tasks.

Tasks	Percentage of time spent (%)
Planning and sequencing	18
Reasoning	11
Concentration	11
Memory	10
Working memory	8
Anticipation	8
Prospective memory	7
Cognitive information speed	7
Social rules	5
Association	5
Cognitive estimation	4

**Table 6:** Summary of neuropsychological test results.

Test	Baseline	T1	T2	Significant differences between the groups
ADAS-Cog	*∅* ± SD	*∅* ± SD	*∅* ± SD	
Group A	21.51 ± 2.27	20.81 ± 2.77	20.5 ± 3.6	
Group B	21.37 ± 4.11	18.33 ± 3.67	18.5 ± 4.2	*P* < 0.001
Group C	22.92 ± 4.02	17.98 ± 2.76	17.4 ± 2.5	
SCOPA-Cog				
Group A	29.07 ± 3.8	27.21 ± 3.6	26.86 ± 3.32	
Group B	29.68 ± 2.87	31.32 ± 3.24	30.71 ± 2.9	*P* < 0.001
Group C	31.83 ± 3.21	39.15 ± 2.9	39.29 ± 2.72	
BADS Zoo (profile)	*∅* ± SD	*∅* ± SD	*∅* ± SD	
Group A	2.5 ± 0.95	3.0 ± 1.2	2.4 ± 1.2	T1: Chi-square: 49.31; *P* < 0.001 T2: Chi-square: 14.42; *P* < 0.001
Group B	2.4 ± 0.9	2.8 ± 1.1	2.6 ± 1.1	
Group C	2.6 ± 0.98	3.54 ± 0.82	3.43 ± 1.0	
BADS instruction	*∅* ± SD	*∅* ± SD	*∅* ± SD	
Group A	2.8 ± 1.3	3.3 ± 1.1	2.9 ± 0.8	T1: Chi-square: 7.1; *P* < 0.03 T2: Chi-square: 9.1; *P* < 0.01
Group B	2.6 ± 1.3	2.9 ± 1.2	3.2 ± 1.1	
Group C	2.7 ± 1.1	3.5 ± 1.1	3.8 ± 0.9	
BADS 6 elements	*∅* ± SD	*∅* ± SD	*∅* ± SD	
Group A	2.8 ± 1.2	3.14 ± 0.89	3.1 ± 0.9	T1: Chi-square: 39.4; *P* < 0.001 T2: Chi-square: 25.3 *P* < 0.01
Group B	2.9 ± 1.2	3.0 ± 1.2	2.9 ± 1.1	
Group C	3.0 ± 0.7	3.55 ± 0.8	3.6 ± 0.9	
PASAT	*∅* ± SD	*∅* ± SD	*∅* ± SD	
Group A	29.94 ± 14.32	32.8 ± 14.83	32.5 ± 13.87	
Group B	31.00 ± 13.32	37.43 ± 12.72	39.57 ± 13.65	*P* < 0.001
Group C	30.4 ± 12.98	46.5 ± 11.5	49.2 ± 13.4	

**Table 7:** Individual goals chosen by the patients and percentage of goal achievement.

Groups	Goal	Goals chosen		Goals obtained		Significance
		Total number	Percentage (%)	Total number	Percentage (%)	
A *N* = 71		*N* = 15	21.1	*N* = 3	20	
B *N* = 75	Dual tasking	*N* = 14	18.7	*N* = 9	64	*P* < 0.001
C *N* = 76		*N* = 15	20	*N* = 10	67	

A *N* = 71		*N* = 15	21.2	*N* = 3	20	
B *N* = 75	Planning of complex tasks	*N* = 16	21.3	*N* = 9	56.3	*P* < 0.001
C *N* = 76		*N* = 17	22.4	*N* = 10	58.9	

A *N* = 71		*N* = 10	14.1	*N* = 4	40	
B *N* = 75	Decision making	*N* = 11	14.7	*N* = 6	54.5	*P* < 0.001
C *N* = 76		*N* = 14	18.4	*N* = 10	71.4	

A *N* = 71		*N* = 13	18.3	*N* = 4	30.8	
B *N* = 75	Rule recognition and rule shifting	*N* = 16	21.3	*N* = 7	43.8	*P* < 0.01
C *N* = 76		*N* = 14	18.4	*N* = 10	71.4	

A *N* = 71		*N* = 12	16.9	*N* = 3	25	
B *N* = 75	Delayed recall	*N* = 12	16	*N* = 7	58.3	*P* < 0.001
C *N* = 76		*N* = 11	14.5	*N* = 9	82	

A *N* = 71		*N* = 6	8.5	*N* = 4	67	
B *N* = 75	Search strategies	*N* = 6	8	*N* = 5	83.3	*P* < 0.01
C *N* = 76		*N* = 5	6.6	*N* = 4	80	

**Table 8:** Effect of the training programme on the main goals (GAS).

GAS		Group A *N* = 71		Group B *N* = 75		Group C *N* = 76	Total	
	Total	Percent	Total	Percent	Total	Percent	Total	Percent
−3	*N* = 12	16.7	*N* = 6	8	*N* = 2	2.6	*N* = 20	8.9
−2	*N* = 10	13.8	*N* = 5	6.7	*N* = 3	3.9	*N* = 18	23.7
−1	*N* = 28	40.3	*N* = 21	28	*N* = 18	23.6	*N* = 67	30
0	*N* = 13	18.1	*N* = 19	25.3	*N* = 23	30.2	*N* = 55	24.7
1	*N* = 8	11.1	*N* = 24	32	*N* = 24	26.3	*N* = 56	25.1
2	*N* = 0	0	*N* = 0	0	*N* = 6	7.9	*N* = 6	2.7
	71		75		76		223	

−3 = worse than start of treatment, −2 = no change, −1 = some improvement, 0 = goal achievement, +1 = slight over-achievement, +2 = great over-achievement.

**Table 9:** UPDRS.

	Group A *N* = 72	Group B *N* = 75	Group C *N* = 76
Baseline			
UPDRS Motor scale	38.56 ± 12.44	37.53 ± 10.76	38.4 ± 11.78
UPDRS Sum Score	59.20 ± 12.4	60.3 ± 12.4	61.5 ± 12.8
Final assessment			
UPDRS Motor scale	34.1 ± 11.4	34.2 ± 11.2	35.2 ± 12.4
UPDRS Sum Score	55.4 ± 12.4	56.3 ± 11.5	57.2 ± 11.4

## References

[1] Chen RC, Chang SF, Su CL (2001). Prevalence, incidence, and mortality of PD: a door-to-door survey in Ilan County, Taiwan. *Neurology*.

[2] Schrag A, Ben-Shlomo Y, Quinn NP (2000). Cross sectional prevalence survey of idiopathic Parkinson’s disease and parkinsonism in London. *British Medical Journal*.

[3] Douglas J, Gelb DJ, Oliver E, Gilman S (1999). Diagnostic criteria for Parkinson disease. *Archives of Neurology*.

[4] Hughes AJ, Daniel SE, Kilford L, Lees AJ (1992). Accuracy of clinical diagnosis of idiopathic Parkinson’s disease: a clinico-pathological study of 100 cases. *Journal of Neurology Neurosurgery and Psychiatry*.

[5] Aarsland D, Andersen K, Larsen JP, Lolk A, Kragh-Sørensen P (2003). Prevalence and characteristics of dementia in Parkinson disease: an 8-year prospective study. *Archives of Neurology*.

[6] Emre M, Aarsland D, Albanese A (2004). Rivastigmine for dementia associated with Parkinson’s disease. *New England Journal of Medicine*.

[7] Levy G, Tang MX, Louis ED (2002). The association of incident dementia with mortality in PD. *Neurology*.

[8] Schrag A, Jahanshahi M, Quinn N (2000). What contributes to quality of life in patients with Parkinson’s disease?. *Journal of Neurology Neurosurgery and Psychiatry*.

[9] Aarsland D, Larsen JP, Karlsen K, Lim NG, Tandberg E (1999). Mental symptoms in Parkinson’s disease are important contributors to caregiver distress. *International Journal of Geriatric Psychiatry*.

[10] Aarsland D, Larsen JP, Tandberg E, Laake K (2000). Predictors of nursing home placement in Parkinson’s disease: a population-based, prospective study. *Journal of the American Geriatrics Society*.

[11] Emre M (2003). Dementia associated with Parkinson’s disease. *Lancet Neurology*.

[12] Braak H, Del Tredici K, Rüb U, De Vos RAI, Jansen Steur ENH, Braak E (2003). Staging of brain pathology related to sporadic Parkinson’s disease. *Neurobiology of Aging*.

[13] Perry EK, Curtis M, Dick DJ (1985). Cholinergic correlates of cognitive impairment in Parkinson’s disease: comparisons with Alzheimer’s disease. *Journal of Neurology Neurosurgery and Psychiatry*.

[14] Litvan I, Aarsland D, Adler CH (2011). MDS task force on mild cognitive impairment in Parkinson’s disease: critical review of PD-MCI. *Movement Disorders*.

[15] Smith EE, Jonides J (1999). Storage and executive processes in the frontal lobes. *Science*.

[16] Burgess PW, Shallice T (1996). Response suppression, initiation and strategy use following frontal lobe lesions. *Neuropsychologia*.

[17] Miller EK, Cohen JD (2001). An integrative theory of prefrontal cortex function. *Annual Review of Neuroscience*.

[18] Abbott RD, White LR, Ross GW, Masaki KH, Curb JD, Petrovitch H (2004). Walking and dementia in physically capable elderly men. *Journal of the American Medical Association*.

[19] Colcombe S, Kramer AF (2003). Fitness effects on the cognitive function of older adults: a meta-analytic study. *Psychological Science*.

[20] Colcombe SJ, Erickson KI, Raz N (2003). Aerobic fitness reduces brain tissue loss in aging humans. *Journals of Gerontology A*.

[21] Colcombe SJ, Erickson KI, Scalf PE (2006). Aerobic exercise training increases brain volume in aging humans. *Journals of Gerontology A*.

[22] Laurin D, Verreault R, Lindsay J, MacPherson K, Rockwood K (2001). Physical activity and risk of cognitive impairment and dementia in elderly persons. *Archives of Neurology*.

[23] Rolland Y, Abellan van Kan G, Vellas B (2010). Healthy brain aging: role of exercise and physical activity. *Clinics in Geriatric Medicine*.

[24] Nelles G (2004). Cortical reorganization—effects of intensive therapy: results from prospective functional imaging studies. *Restorative Neurology and Neuroscience*.

[25] Shepherd RB (2001). Exercise and training to optimize functional motor performance in stroke: driving neural reorganization?. *Neural Plasticity*.

[26] Kramer AF, Hahn S, Cohen NJ (1999). Ageing, fitness and neurocognitive function. *Nature*.

[27] Baatile J, Langbein WE, Weaver F, Maloney C, Jost MB (2000). Effect of exercise on perceived quality of life of individuals with Parkinson’s disease. *Journal of Rehabilitation Research and Development*.

[28] Reuter I, Engelhardt M, Stecker K, Baas H (1999). Therapeutic value of exercise training in Parkinson’s disease. *Medicine and Science in Sports and Exercise*.

[29] Dujardin K, Duhamel A, Delliaux M, Thomas-Antérion C, Destée A, Defebvre L (2010). Cognitive complaints in Parkinson’s disease: its relationship with objective cognitive decline. *Journal of Neurology*.

[30] Fahn S, Elton RL, The Members of the UPDRS Development Committee, Fahn S, Marsden CD, Goldstein M (1987). Unified Parkinson’s disease rating scale. *Recent Developments in Parkinson’s Disease II*.

[31] Royal College of Physicians and the Intercollegiate Stroke Working Party (2008). *National Clinical Guideline for Stroke*.

[32] Kalbe E, Calabrese P, Kohn N (2008). Screening for cognitive deficits in Parkinson’s disease with the Parkinson neuropsychometric dementia assessment (PANDA) instrument. *Parkinsonism and Related Disorders*.

[33] Folstein MF, Folstein SE, McHugh PR (1975). ’Mini mental state’. A practical method for grading the cognitive state of patients for the clinician. *Journal of Psychiatric Research*.

[34] Rosen WG, Mohs RC, Davis KL (1984). A new rating scale for Alzheimer’s disease. *American Journal of Psychiatry*.

[35] Talwalker S (1996). Assessment of AD with the ADAS-cog. *Journal of Geriatric Psychiatry and Neurology*.

[36] Marinus J, Visser M, Verwey NA (2003). Assessment of cognition in Parkinson’s disease. *Neurology*.

[37] Gronwall DMA (1977). Paced auditory serial addition task: a measure of recovery from concussion. *Perceptual and Motor Skills*.

[38] Wilson BA, Evans JJ, Emslie H, Alderman N, Burgess P (1998). The development of an ecologically valid test for assessing patients with a dysexecutive syndrome. *Neuropsychological Rehabilitation*.

[39] Lehrl S (1989). *Mehrfach-Wortschatz-Intelligenztest: MWT-B*.

[40] Zigmond AS, Snaith RP (1983). The hospital anxiety and depression scale. *Acta Psychiatrica Scandinavica*.

[41] Jenkinson C, Peto V, Fitzpatrick R, Greenhall R, Hyman N (1995). Self-reported functioning and well-being in patients with Parkinson’s disease: comparison of the short-form wealth survey (SF-36) and the Parkinson’s disease questionnaire (PDQ-39). *Age and Ageing*.

[42] Lewis SJG, Slabosz A, Robbins TW, Barker RA, Owen AM (2005). Dopaminergic basis for deficits in working memory but not attentional set-shifting in Parkinson’s disease. *Neuropsychologia*.

[43] Fournet N, Moreaud O, Roulin JL, Naegele B, Pellat J (2000). Working memory functioning in medicated Parkinson’s disease patients and the effect of withdrawal of dopaminergic medication. *Neuropsychology*.

[44] Golubović S, Tubić T, Marković S (2011). Psycho-motor re-education-movement as therapeutic method. *Medicinski Pregled*.

[45] Golubović T, Maksimović J, Golubović B, Glumbić N (2012). Effects of exercise on physical fitness in children with intellectual disability. *Research in Developmental Disabilities*.

[46] Oswald WD, Rupprecht R, Gunzelmann T, Tritt K (1996). The SIMA-project: effects of 1 year cognitive and psychomotor training on cognitive abilities of the elderly. *Behavioural Brain Research*.

[47] Oswald WD, Gunzelmann T, Rupprecht R, Hagen B (2006). Differential effects of single versus combined cognitive and physical training with older adults: the SimA study in a 5-year perspective. *European Journal of Ageing*.

[48] Goebel S, Mehdorn HM, Leplow B (2010). Strategy instruction in Parkinson’s disease: influence on cognitive performance. *Neuropsychologia*.

[49] Baddeley AD (1986). *Working Memory*.

[50] Norman DA, Shallice T, Gazzaniga MS (2000). (1980) Attention to action: willed and automatic control of behaviour. *Cognitive Neuroscience A*.

[51] Sammer G, Reuter I, Hullmann K, Kaps M, Vaitl D (2006). Training of executive functions in Parkinson’s disease. *Journal of the Neurological Sciences*.

[52] Churchill JD, Galvez R, Colcombe S, Swain RA, Kramer AF, Greenough WT (2002). Exercise, experience and the aging brain. *Neurobiology of Aging*.

[53] Klepac N, HajnÅek S, Trkulja V (2010). Cognitive performance in nondemented nonpsychotic parkinson disease patients with or without a history of depression prior to the onset of motor symptoms. *Journal of Geriatric Psychiatry and Neurology*.

[54] Hullmann K, Sammer G, Reuter I Training of executive functions in Parkinson’s disease.

[55] París AP, Saleta HG, de la Cruz Crespo Maraver M (2011). Blind randomized controlled study of the efficacy of cognitive training in Parkinson’s disease. *Movement Disorders*.

[56] Sinforiani E, Banchieri L, Zucchella C, Pacchetti C, Sandrini G (2004). Cognitive rehabilitation in Parkinson’s disease.. *Archives of Gerontology and Geriatrics*.

[57] Hausdorff JM, Yogev G, Springer S, Simon ES, Giladi N (2005). Walking is more like catching than tapping: gait in the elderly as a complex cognitive task. *Experimental Brain Research*.

[58] Yogev G, Giladi N, Peretz C, Springer S, Simon ES, Hausdorff JM (2005). Dual tasking, gait rhythmicity, and Parkinson’s disease: which aspects of gait are attention demanding?. *European Journal of Neuroscience*.

